# Supplementary material for: Surfactant-Free Synthesis of Three-Dimensional Perovskite Titania-Based Micron-Scale Motifs Used as Catalytic Supports for the Methanol Oxidation Reaction
Source: Molecules. 2021 Feb 9;26(4):909. doi: 10.3390/molecules26040909 (PMC7915617; doi:10.3390/molecules26040909)
Supplement: Supplementary file 1 [file molecules-26-00909-s001.pdf]

Supplementary Information for

"Surfactant-free Synthesis of Three-dimensional Perovskite Titania-based Micron-scale Motifs Used as Catalytic Supports for the Methanol Oxidation Reaction"

Nathaniel Hurley,[1] Luyao Li,[1] Christopher Koenigsmann,[2] and Stanislaus S. Wong[1,*]

Email: stanislaus.wong@stonybrook.edu; sswong@bnl.gov

[1]Department of Chemistry, State University of New York at Stony Brook,

Stony Brook, NY 11794-3400

[2]Department of Chemistry, Fordham University,

Bronx, NY 10458

*To whom correspondence should be addressed.

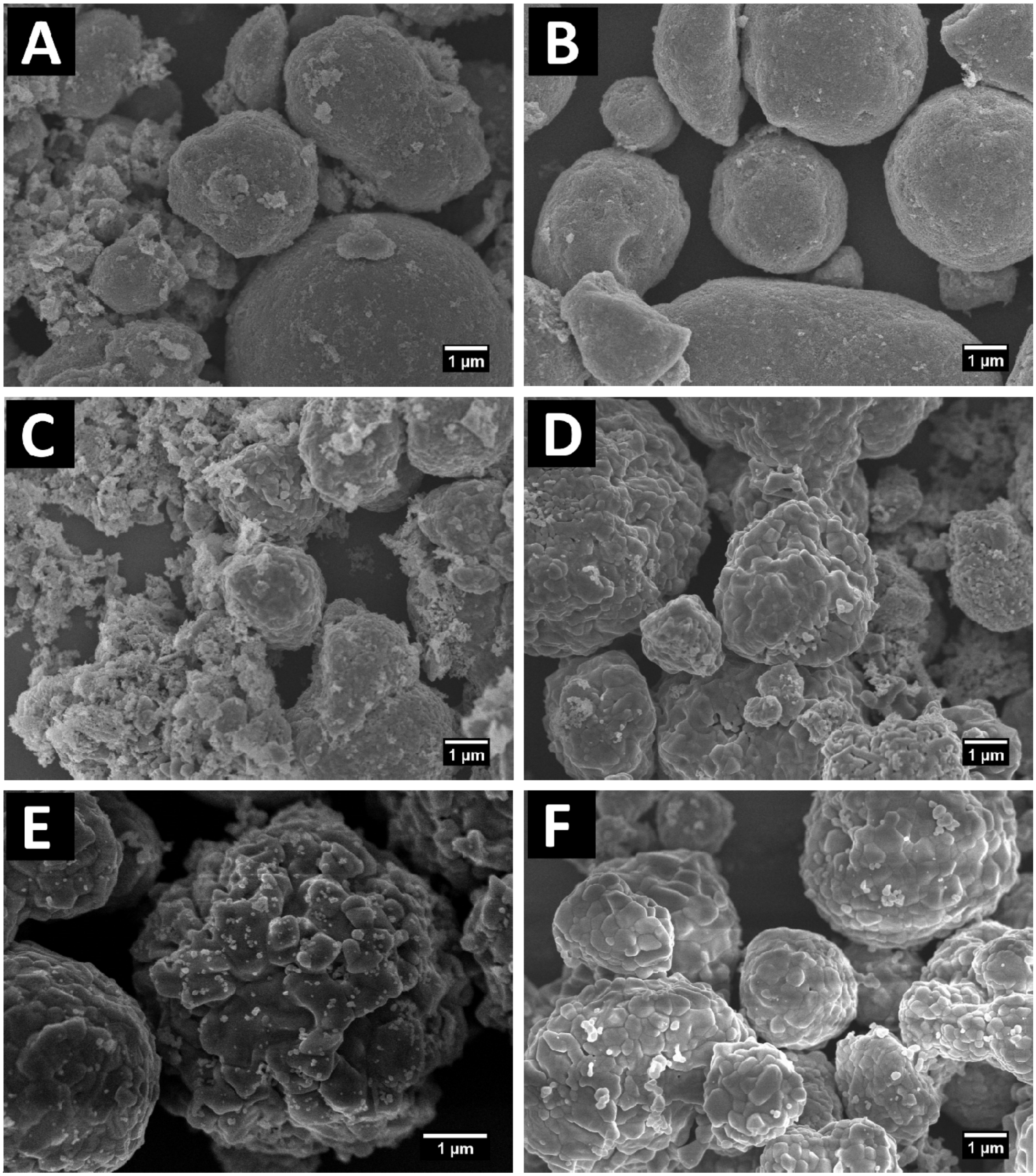

Figure S1. SEM images of CTO, annealed at various temperatures, including (A) 600°C, (B) 700°C, (C) 800°C, (D) 900°C, (E) 1000°C, and (F) 1100°C, respectively.

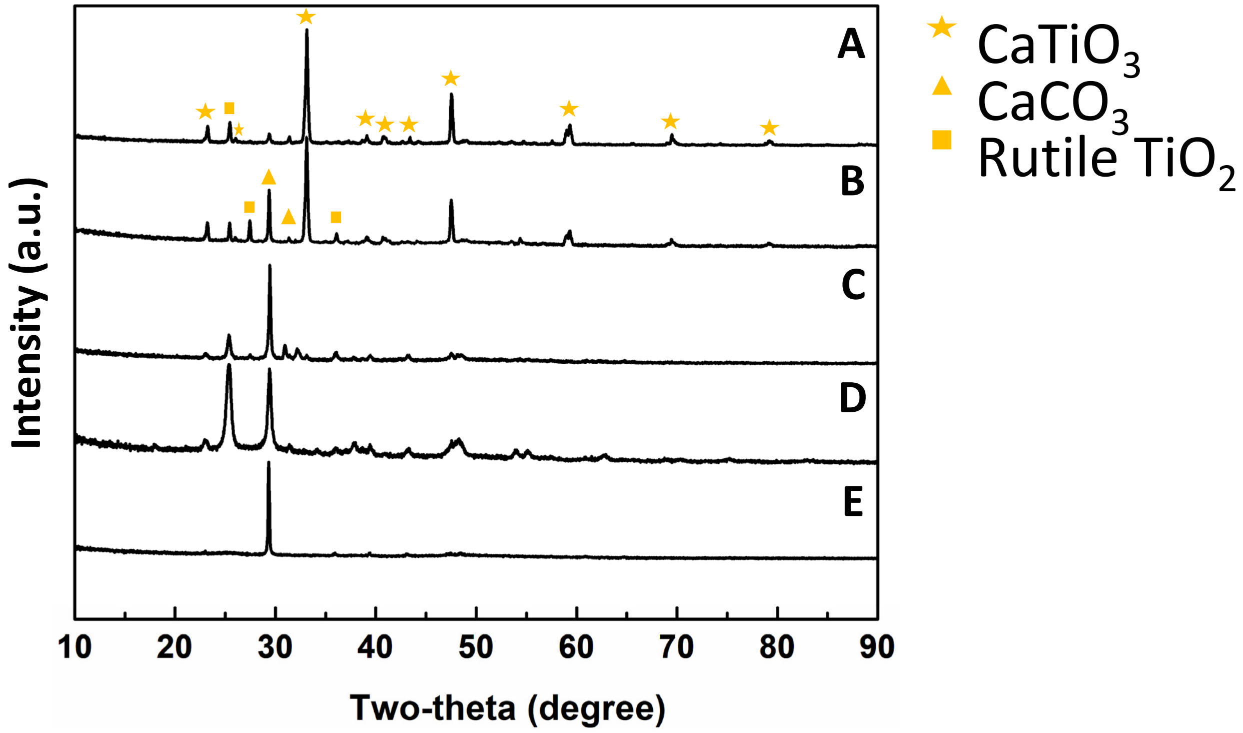

Figure S2. XRD patterns of a CTO powder sample calcined at (A) 1100ºC; (B) 1000ºC; (C) 800ºC; and (D) 600ºC, respectively, as well as of the (E) CTO intermediate. The data on all samples were acquired without an acid wash.

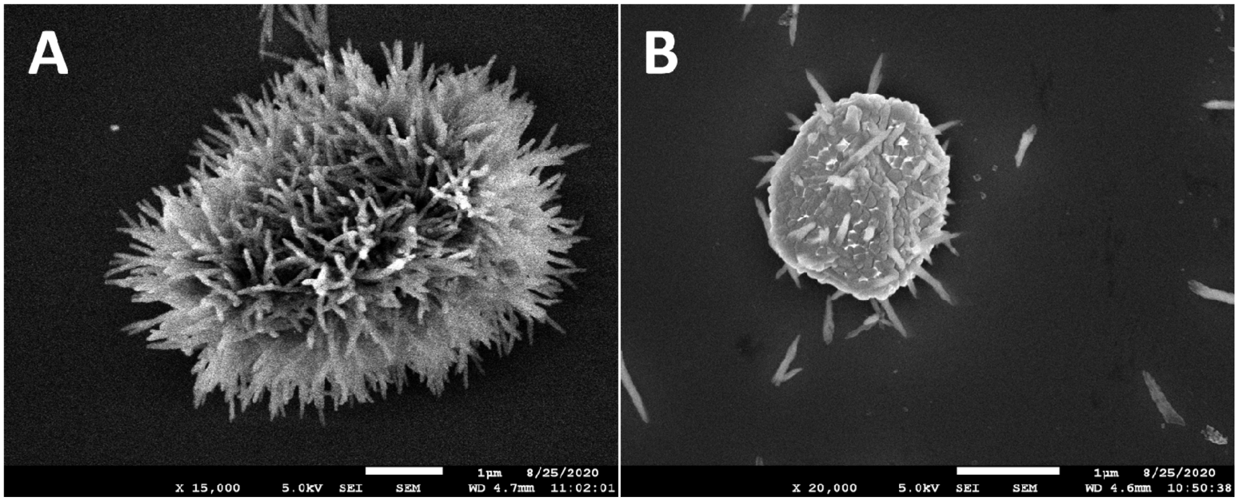

Figure S3. SEM images of TiO_2 rod-like impurities and "sea urchin" motifs within isolated (A) CTO and (B) STO samples, respectively, prior to the 'nitric acid' wash.

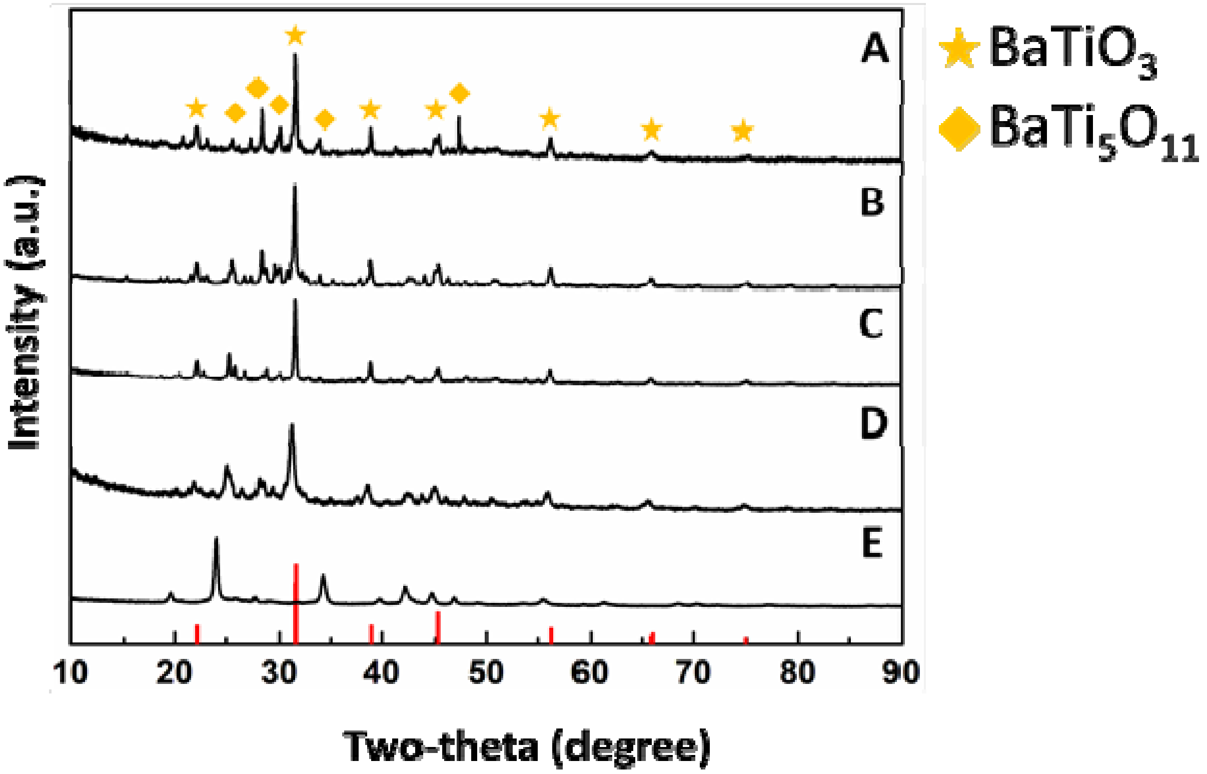

Figure S4. XRD patterns of a BTO powder sample, calcined at (A) 1100ºC, (B) 1000ºC, (C) 900ºC, and (D) 800ºC, respectively, as well as of the (E) BTO intermediate. The data on all samples were obtained without an acid wash. Triangles indicate the presence of $BaTi_5O_{11}$ peaks.

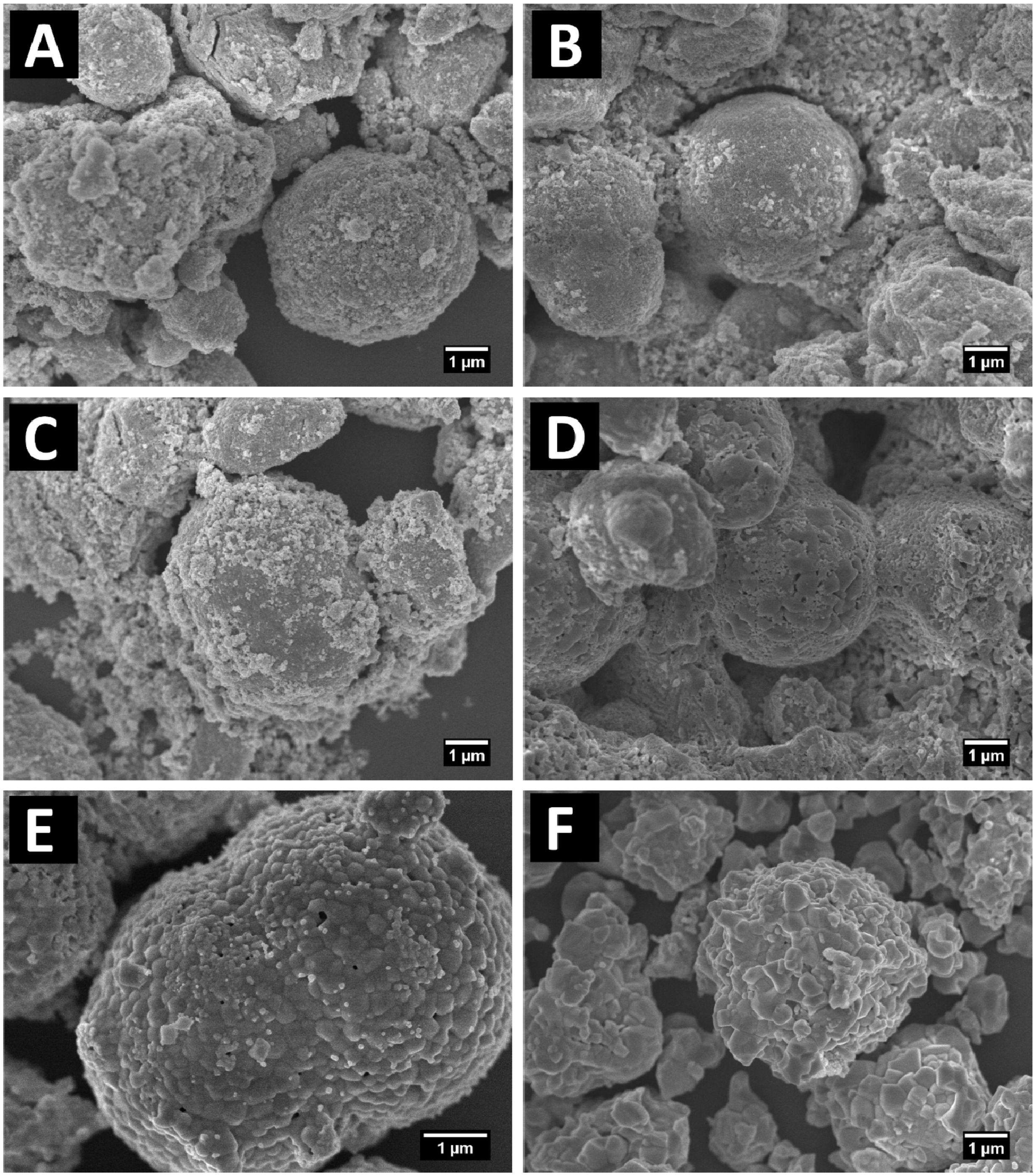

Figure S5. SEM images of BTO, annealed at various temperatures, including (A) 600°C, (B) 700°C, (C) 800°C, (D) 900°C, (E) 1000°C, and (F) 1100°C, respectively.

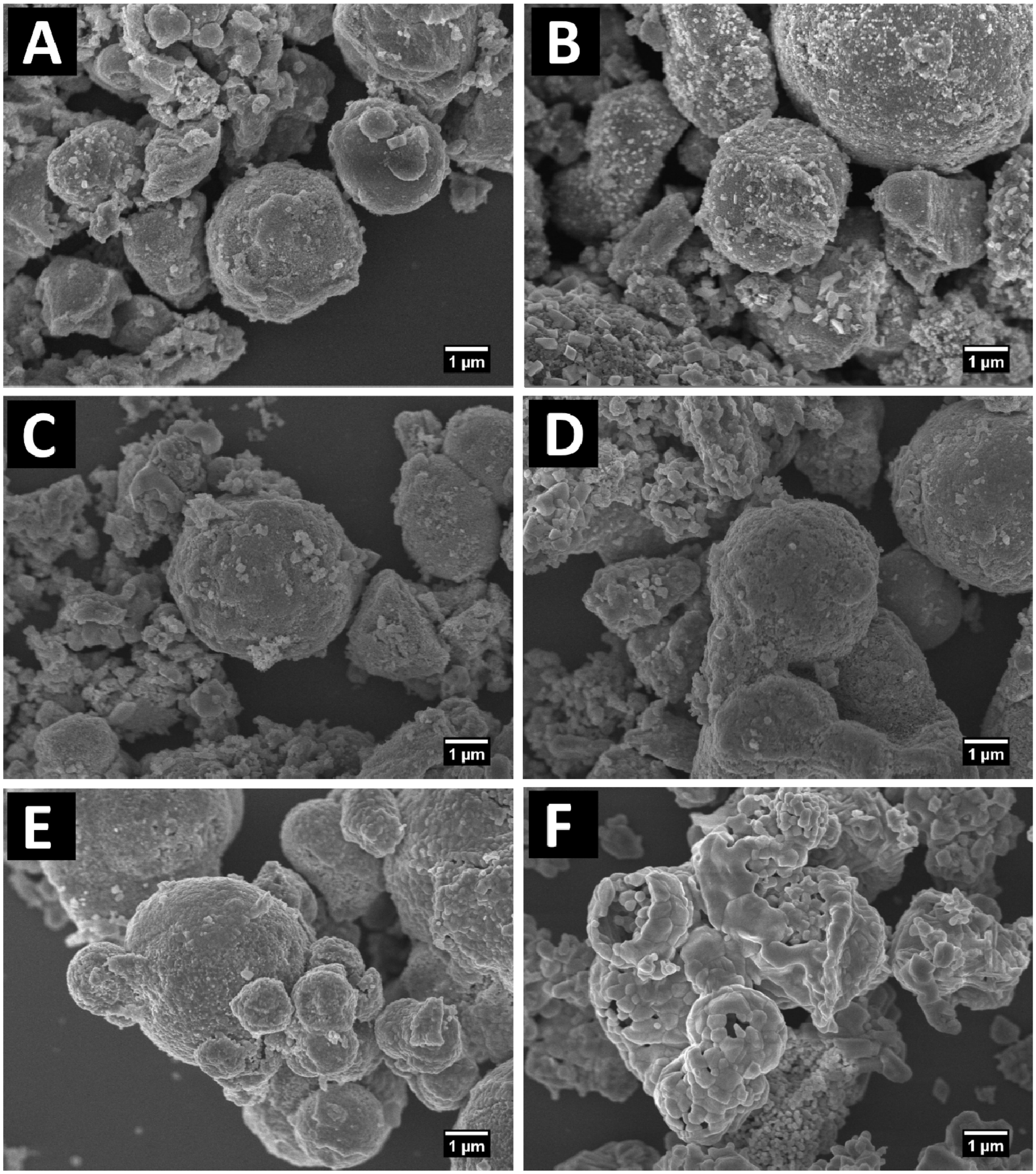

Figure S6. SEM images of STO annealed at various temperatures, including (A) 600°C, (B) 700°C, (C) 800°C, (D) 900°C, (E) 1000°C, and (F) 1100°C, respectively.

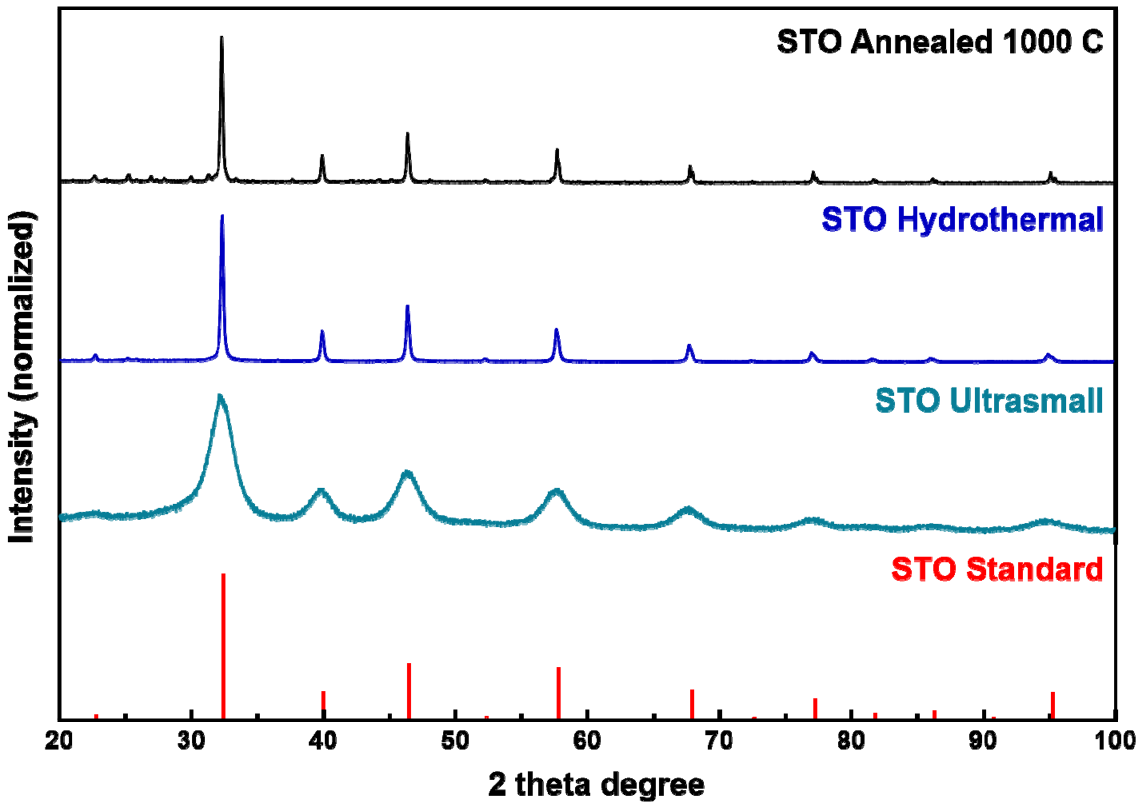

Figure S7. XRD patterns of annealed, hydrothermal-derived, and ultra-small STO samples as compared with the standard STO reference pattern.

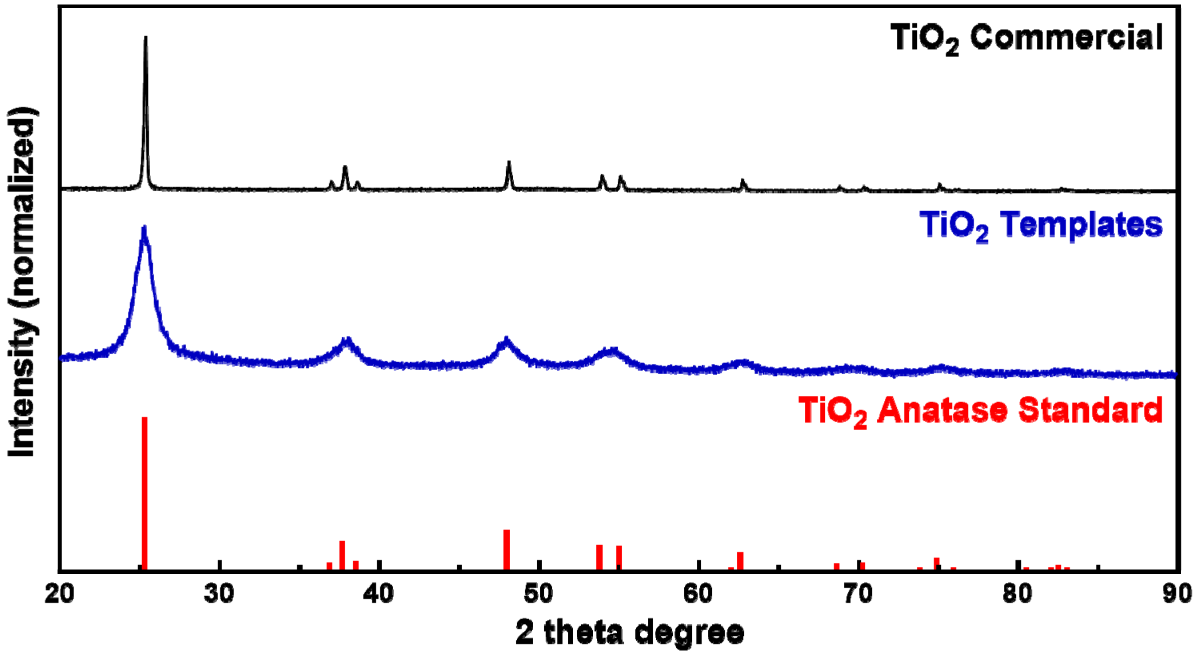

Figure S8. XRD patterns of the TiO_2 3D precursor templates along with the commercial TiO_2 nanoparticles, with both samples compared with respect to the anatase TiO_2 database standard.

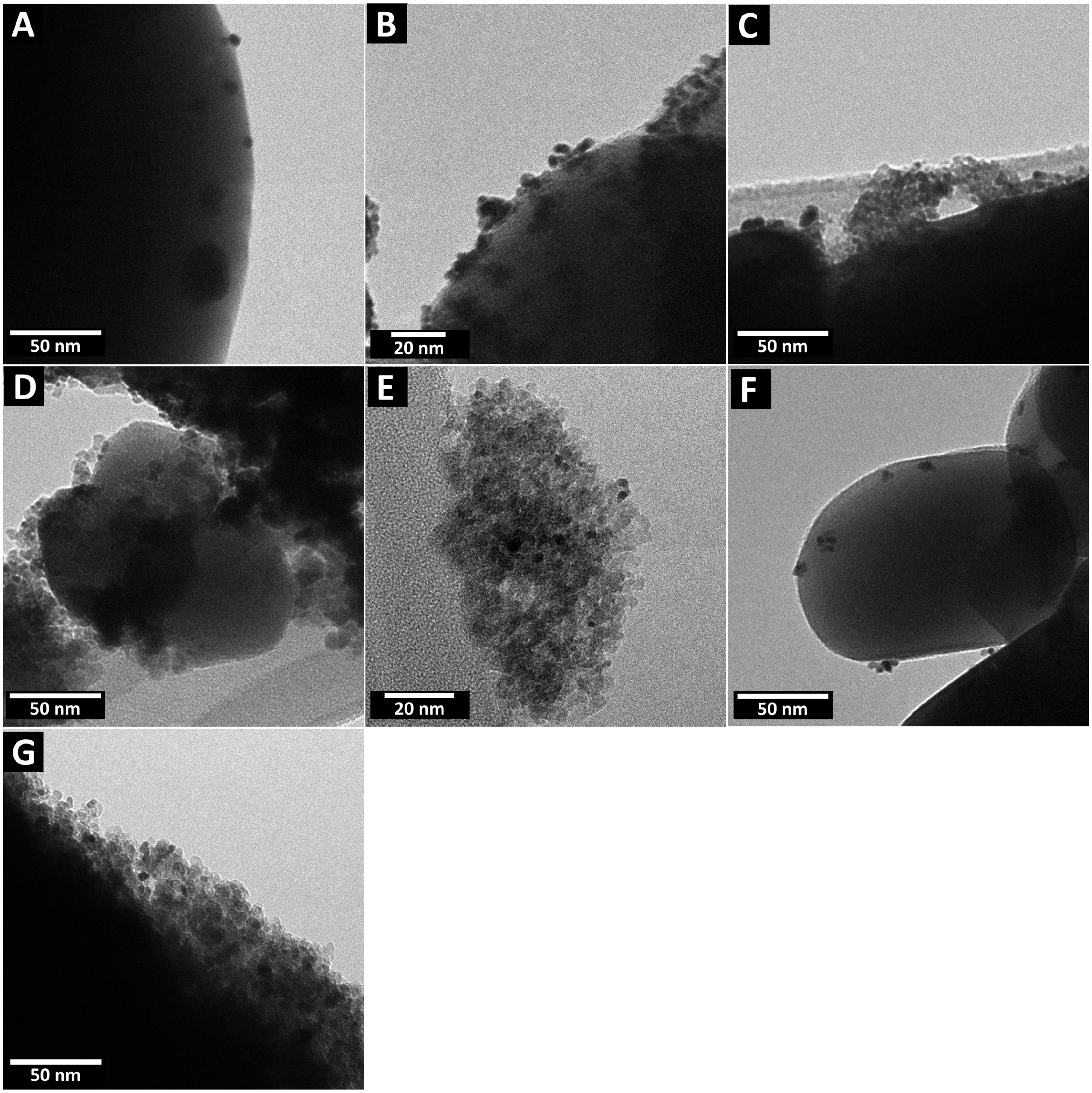

Figure S9. TEM images of Pt particles deposited onto the various different perovskite and standard samples that were analyzed. These systems include (A) Pt/CTO, (B) Pt/STO, (C) Pt/BTO, (D) Pt/STO hydrothermal, (E) Pt/STO ultra-small, (F) Pt/TiO_2 commercial, and (G) Pt/TiO_2 precursor templates, respectively.

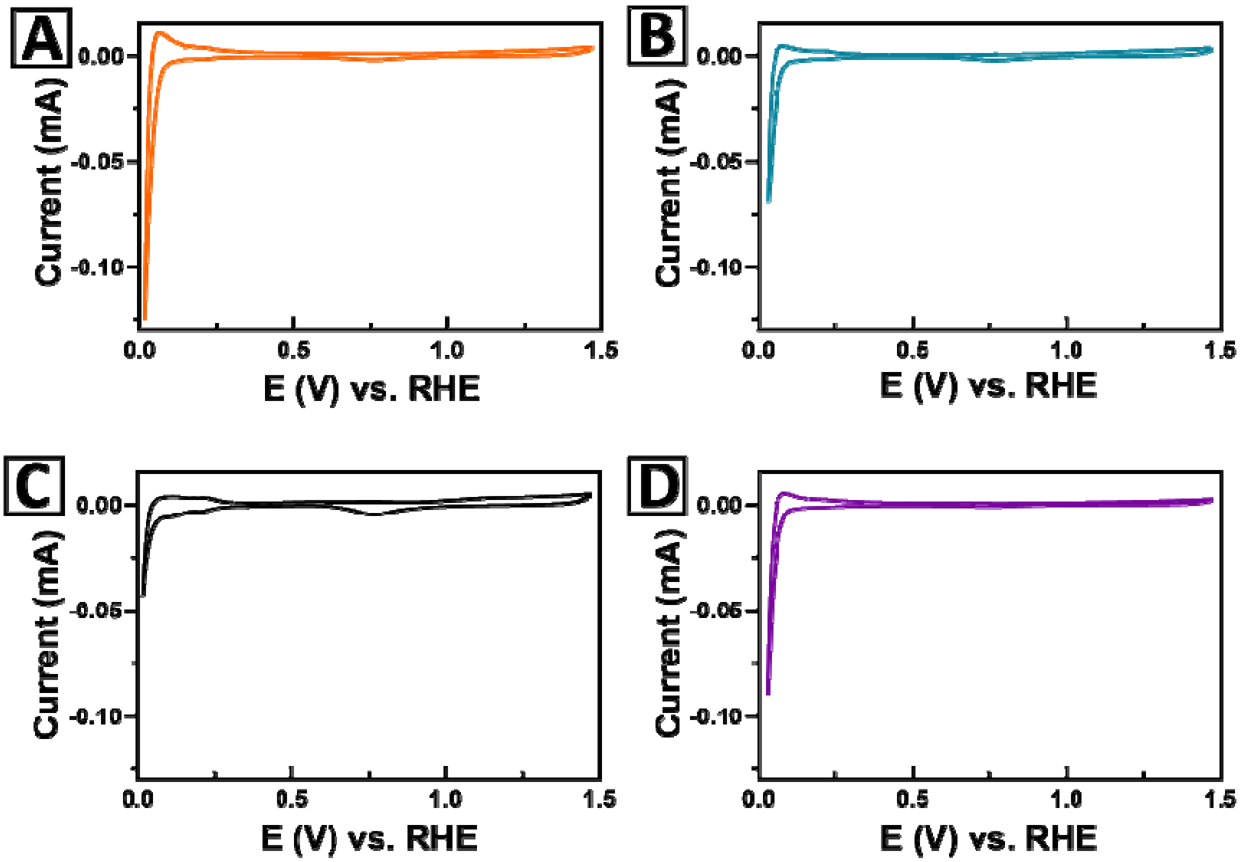

Figure S10. CV curves for reference standards of (A) Pt/STO hydrothermal, (B) Pt/STO ultra-small, (C) Pt/TiO_2 commercial, and (D) Pt/TiO_2 template systems, respectively

**Material System**	**Specific activity (mA / cm^2)**		**Mass Activity (mA / mg)**		**Specific Surface Area (m^2 / g)**
	**0.7 V**	**0.8 V**	**0.7 V**	**0.8 V**	
Pt / CTO - annealed	0.033	0.151	1.920	8.780	5.80
Pt / STO - annealed	0.030	0.138	1.150	5.300	3.85
Pt / STO - hydrothermal	0.016	0.030	0.103	0.198	0.65
Pt / STO (ultra-small)	0.018	0.032	0.056	0.099	0.31
Pt / BTO - annealed	0.021	0.114	1.200	6.420	5.70
Pt / TiO_2 templates	0.008	0.012	0.024	0.035	0.28
Pt / TiO_2 (commercial)	0.033	0.104	0.197	0.624	0.60

Table S1. MOR data obtained for all samples, including specific and mass activity readings, in addition to specific surface area values.

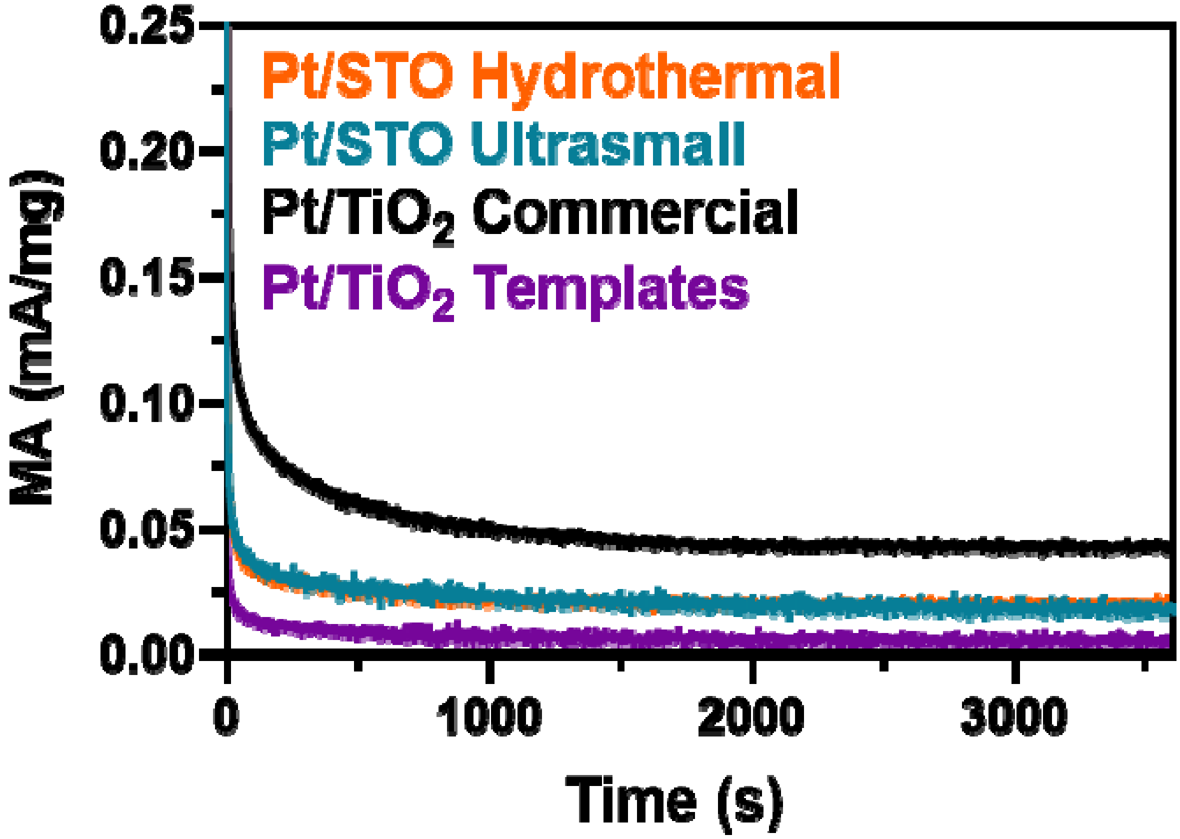

Figure S11. Chronoamperometry measurements collected at 0.8 V of reference standard systems associated with Pt/STO hydrothermal (orange), Pt/STO ultra-small (teal), Pt/TiO_2 commercial (black), and Pt/TiO_2 templates (purple), respectively, obtained within an Ar-saturated 0.1 M perchloric acid solution + 0.5 M MeOH medium for 3600 sec.
